# Evolution of the Symbiosis-Specific GRAS Regulatory Network in Bryophytes

**DOI:** 10.3389/fpls.2018.01621

**Published:** 2018-11-06

**Authors:** Christopher Grosche, Anne Christina Genau, Stefan A. Rensing

**Affiliations:** ^1^Plant Cell Biology, Faculty of Biology, University of Marburg, Marburg, Germany; ^2^BIOSS Centre for Biological Signalling Studies, University of Freiburg, Freiburg, Germany

**Keywords:** bryophyte, land plant evolution, moss, mycorrhiza, *Physcomitrella patens*, symbiotic pathway, GRAS, transcription factor

## Abstract

Arbuscular mycorrhiza is one of the most common plant symbiotic interactions observed today. Due to their nearly ubiquitous occurrence and their beneficial impact on both partners it was suggested that this mutualistic interaction was crucial for plants to colonize the terrestrial habitat approximately 500 Ma ago. On the plant side the association is established via the common symbiotic pathway (CSP). This pathway allows the recognition of the fungal symbiotic partner, subsequent signaling to the nucleus, and initiation of the symbiotic program with respect to specific gene expression and cellular re-organization. The downstream part of the CSP is a regulatory network that coordinates the transcription of genes necessary to establish the symbiosis, comprising multiple GRAS transcription factors (TFs). These regulate their own expression as an intricate transcriptional network. Deduced from non-host genome data the loss of genes encoding CSP components coincides with the loss of the interaction itself. Here, we analyzed bryophyte species with special emphasis on the moss *Physcomitrella patens*, supposed to be a non-host, for the composition of the GRAS regulatory network components. We show lineage specific losses and expansions of several of these factors in bryophytes, potentially coinciding with the proposed host/non-host status of the lineages. We evaluate losses and expansions and infer clade-specific evolution of GRAS TFs.

## Introduction

Mycorrhiza is the most common plant–fungus symbiotic interaction we observe today. Over 80% of all extant plant species engage in this symbiotic interaction (Bonfante and Genre, 2010) which is beneficial to both partners (mutualistic). The plant provides the fungus with carbohydrates and lipids, in turn the fungus provides the plant host with nutrients like nitrate and especially phosphorus. Additionally, the fungal hyphae enlarge the rhizosphere area of the plant and seem to improve plant stress tolerance (Bago et al., 2003; Liu et al., 2007; Feddermann et al., 2010; Veresoglou et al., 2012). Several forms of mycorrhizal interactions exist, of which arbuscular mycorrhiza (AM) is the most common one. It is called ‘arbuscular’ since the fungal hyphae grow into the plant cells forming a ‘tree-like’ structure called arbuscule. In AM this structure represents the nutrient exchange zone between plant and fungus, since the plant-derived so-called peri-arbuscular membrane is loaded with transporters to facilitate the described nutrient exchange. The actual composition of the respective membrane in terms of transporters is predominantly known for plants (Harrison et al., 2002; Bonfante and Genre, 2010; Gaude et al., 2012; Luginbuehl and Oldroyd, 2017; MacLean et al., 2017).

**FIGURE 1:**
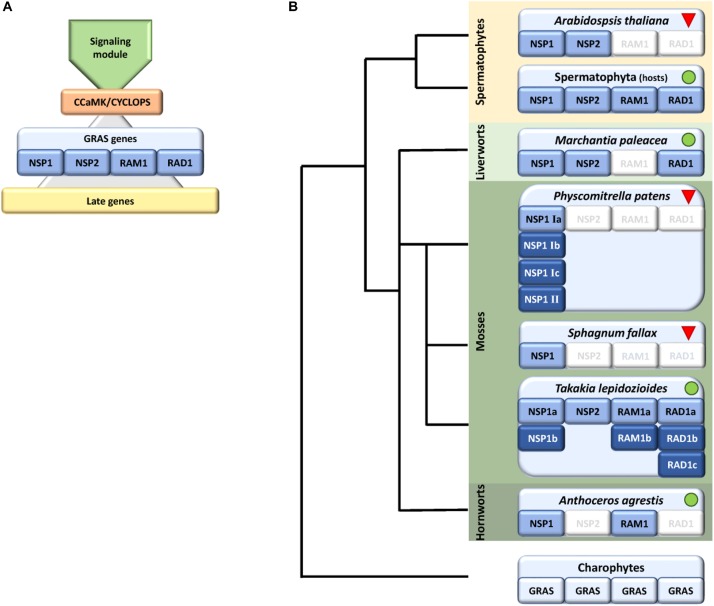
Schematic representation of identified factors in the symbiosis GRAS signaling module with a focus on bryophytes. **(A)** Scheme showing the main parts of the common symbiotic pathway (CSP), consisting of a signaling module, the main hub CCaMK/CYCLOPS, which controls start of the transcriptional program eventually controlled by GRAS genes (NSP1/2, RAD1, and RAM1). The GRAS module regulates the transcription of later genes. **(B)** Presence/absence of GRAS factors within bryophytes shown on a schematic tree, predominantly based on genomic data. We were not able to identify RAM1/RAD1 in liverworts and NSP2/RAD1 in hornworts, with the exception of RAD1 in transcriptomic data of *M. paleacea*. In case of mosses we observed an expansion of NSP1 in the crown group mosses (Bryophytina) but only *Takakia* shows the full set of symbiotic GRAS genes. *Sphagnum* encodes NSP1 and NSP2 whereas crown group (“true”) mosses, exemplified by *P. patens*, encode up to six NSP1 genes. Dark blue coloring indicates duplications of respective genes. Green dots and red triangles indicate known host and non-host status, respectively. The land plant ancestor most probably encoded all four symbiosis-specific GRAS sub families.

Although beneficial for them, plants need to regulate and coordinate this symbiotic interaction because intensive cellular reprogramming is required and the plant needs to restrict the degree of colonization by the fungus in correspondence to its own nutritional status (Koide and Schreiner, 1992; Breuillin et al., 2010; Balzergue et al., 2011), e.g., to avoid carbon loss (Carbonnel and Gutjahr, 2014). Additionally, and perhaps most important, the beneficial partner needs to be distinguished from potential pathogens. Plants, and most probably already their progenitors, the streptophyte algae, evolved the so called common symbiotic pathway (CSP) (Oldroyd, 2013; Delaux et al., 2015) that enables this distinctive signaling. The pathway is called ‘common’ because a large part of the set of genes that evolved to accommodate arbuscular mycorrhiza (AM) was later recruited by the *Rhizobium* legume symbiosis (Kistner and Parniske, 2002). Numerous components of the CSP in AM host plants have been identified, but these analyzes were predominantly performed in seed plants (see Delaux et al., 2013b; Oldroyd, 2013 for review and Figure 1A for overview). Via this pathway the plant detects the nearby fungus by its secreted lipo-chito-oligosaccharides (LCOs) and other myc factors such as short-chain chitin oligomers (COs) (Maillet et al., 2012; Genre et al., 2013) and prepares for colonization by starting a specific transcriptional program and cellular reorganization (Gutjahr and Parniske, 2013; Pimprikar and Gutjahr, 2018). In turn, the fungus senses the plant host, predominantly through strigolactones secreted by the plant, and starts intensive hyphal growth and branching toward the symbiotic partner (Akiyama et al., 2005; Besserer et al., 2006). The fungal signals are recognized by a receptor complex at the plant plasma membrane that involves Lysine motif (LysM) receptor like kinases (RLKs). This complex seems to be more intricate than previously thought, since it becomes more and more evident that multiple signals and receptors contribute to composite signal processing (Antolín-Llovera et al., 2012; Conn and Nelson, 2015; Gutjahr et al., 2015; Sun et al., 2015; Zhang et al., 2015). The signal is transduced to the nucleus by a so far not fully characterized mechanism involving most probably mevalonate and potentially further factors (Venkateshwaran et al., 2015). Multiple ion channels in the nuclear envelope elicit a symbiotic Ca^2+^ oscillating signal (spiking) in the nucleus (Charpentier et al., 2008). The factors described so far make up what we will henceforth call the ‘signaling module’ of the CSP (Figure 1A). This module transduces the external signal to the nucleus where it results in calcium oscillation. This symbiosis-specific calcium spiking activates the calcium and calmodulin-dependent kinase (CCaMK), a key player of the CSP, which in turn regulates the transcription factor (TF) CYCLOPS, which is thought to initiate a transcriptional regulatory network (Singh et al., 2014;Pimprikar et al., 2016). This network of various TFs controls, together with CYCLOPS, the transcription of some additional TFs and the ‘later genes’ that encode factors which are, for example, needed for arbuscule initiation, branching and transmembrane transport (Harrison et al., 2002; Zhang et al., 2010; Takeda et al., 2011). The transcription of all those factors is tightly regulated and especially GRAS [Gibberellic acid insensitive (GAI), Repressor of GAI (RGA), and Scarecrow (SCR)] proteins are important regulators in this developmental process (Gutjahr, 2014; Xue et al., 2015). This family originated from a bacterial methylase (Zhang et al., 2012) and apparently evolved in streptophyte algae (Wilhelmsson et al., 2017), the sister lineage to land plants. GRAS proteins fulfill important regulatory roles in plant growth, response to environment and development (Peng et al., 1999; Pysh et al., 1999; Hirsch and Oldroyd, 2009; Sun et al., 2011). Recently the DNA binding capability of GRAS proteins was reported (Li et al., 2016), demonstrating that they might act as TFs. However, their mode of action as regulators is still highly debated (Hirano et al., 2017). In case of arbuscular mycorrhiza, so far, predominantly Reduced Arbuscular Mycorrhization 1 (RAM1), Required for Arbuscule Development1 (RAD1), Nodulation signaling pathway 1 (NSP1) and NSP2 were identified as prominent regulators, although NSP1 and NSP2 were previously thought to be root nodule symbiosis specific (Gobbato et al., 2012, 2013; Lauressergues et al., 2012; Maillet et al., 2012; Delaux et al., 2013a; Hohnjec et al., 2015; Park et al., 2015; Rich et al., 2015; Xue et al., 2015; Pimprikar et al., 2016) (Figure 1A). Additionally, recently further potential GRAS TFs were proposed to be involved in mycorrhizal regulation (Xue et al., 2015; Heck et al., 2016). It has been suggested that the action of the four mentioned GRAS TFs is highly interconnected or dependent of each other, thus forming a transcriptional network (Xue et al., 2015). For instance, it was shown that RAM1 interacts with RAD1 and controls several ‘later genes’ (Park et al., 2015; Xue et al., 2015). The transcription of RAM1 in turn is controlled by CYCLOPS and DELLA (Pimprikar et al., 2016). NSP1 and NSP2 were shown to interact directly in nodulation (Hirsch et al., 2009). Additionally, NSP2 was shown to interact with RAM1 by yeast-2-hybrid and bimolecular fluorescence complementation (Gobbato et al., 2012). Adding an additional layer of complexity in the control of this symbiosis, it was shown that NSP2 is regulated by the microRNA MiR171h in flowering plants (Devers et al., 2011; Lauressergues et al., 2012; Hofferek et al., 2014).

Due to their structure and mode of action, e.g., being functional as homo- or hetero-dimer, GRAS TFs seem not only to act as TFs but also as some kind of ‘hub proteins’ to interconnect signals from different pathways, e.g., hormone signaling, to regulate complex cellular reprogramming (Thieulin-Pardo et al., 2015; Li et al., 2016). This hub function becomes obvious in the example of DELLA proteins. These proteins are specialized GRAS TFs consisting of a GRAS domain and an additional DELLA domain. These are known to be key regulators in gibberellic acid (GA) signaling (Sun, 2011). GA presence inhibits arbuscule formation, and DELLA proteins are degraded under this condition. In turn, DELLA proteins, although not AM specific, are important for arbuscule formation (Floss et al., 2013). Very recently it was shown that DELLAs interact with CCaMK/CYCLOPS and potentially additional TFs to regulate RAM1 transcription (Pimprikar et al., 2016). Additionally, DELLAs interact with the GRAS TF DELLA interacting protein 1 (DIP1) and RAD1, which in turn interact with RAM1 (Yu et al., 2014; Park et al., 2015; Takeda et al., 2015; Xue et al., 2015; Pimprikar et al., 2016), indicating the importance of DELLA and plant hormones in AM development and regulation. Moreover, abscisic acid (ABA) has been shown to promote mycorrhizal development, possibly by stabilizing DELLA (Achard et al., 2006), and by regulating GA levels in the context of symbiosis (Martin-Rodriguez et al., 2016).

Recently, the evolution of the CSP was analyzed, covering datasets ranging from chlorophytes to spermatophytes (Delaux et al., 2014, 2015). It was shown that CSP factors are present in charophyte algae, especially those for signal perception and processing of the Ca^2+^ signal in the nucleus. Hence, some CSP factors were already present before the water-to-land transition (Delaux et al., 2015). However, with respect to the GRAS genes, orthologs of the symbiosis-specific GRAS TFs known from extant land plants were not detected (Delaux et al., 2015).

Symbiosis specific genes are lost in non-host plants, leading to a specific absence/presence pattern of CSP components (Delaux et al., 2014; Favre et al., 2014; Bravo et al., 2016). Hence, presence and absence of these factors may allow a conclusion on the symbiotic status of the plant analyzed. Indeed, some land plant lineages lost the ability to form a mycorrhizal partnership, among them the Brassicaceae with the prime plant model, the weed *Arabidopsis thaliana*. The model moss *Physcomitrella patens* (Funariceae) is not known to form AMF associations in nature, although intracellular growth can be occasionally detected in culture (Hanke and Rensing, 2010) and the relative *Funaria hygrometrica* was described to show AMF association in a companion plant assay (Parke and Linderman, 1980). While *A. thaliana* has lost genes required for responding to symbiotic fungi (Delaux et al., 2014), *P. patens* retained orthologs of these, at least for factors of the signaling module of the CSP (Wang et al., 2010; Delaux et al., 2015). Our study focuses on bryophytes, comprising mosses, hornworts, and liverworts. While liverworts and hornworts are generally considered host plants, most mosses are considered non-hosts (Field and Pressel, 2018). The crown group mosses (Bryophytina or true mosses) comprise the classes Oedipodiopsida, Polytrichopsida, Tetraphidopsida (each with a single sub class) as well as the major class Bryopsida, comprising eight sub classes. Sister lineages to the Bryophytina are the three single class comprising sub divisions Andreaeophytina, Sphagnophytina (comprising the genus *Sphagnum*, peat mosses) and Takakiophytina. Their branching order remains under debate, with Takakiophytina (comprising the single genus *Takakia* with the two species *T. lepidoziodes* and *T. ceratophylla*) probably being sister to all other mosses (Volkmar and Knoop, 2010; Ligrone et al., 2012). The only accepted evidence for host plants within the mosses is in the basal lineage represented by *Takakia* (Boullard, 1988). Here, we performed comprehensive phylogenomic analyzes of the GRAS transcriptional regulatory network in bryophytes and found lineage specific losses and expansions of these key symbiotic signaling pathway components. We evaluate our findings with respect to the host and non-host status of the species or lineages in question and hypothesize on the (early) clade-specific evolution of symbiotic GRAS genes.

## Materials and Methods

### Phylogenetic Analyses

GRAS TFs were acquired using the HMM-based (using the motif PF03514) TAPscan classification (Wilhelmsson et al., 2017) against a database of sequenced plant and algal genomes and transcriptomes (Supplementary Table S1). An initial alignment and phylogenetic tree of all GRAS TFs was constructed, and sequences from the clades representing the sub families involved in symbiosis signaling (NSP1, NSP2, RAD1, RAM1) were selected, aligned, manually curated and used to generate HMMs specific to each of the four sub families using hmmbuild from HMMer (Finn et al., 2015) 3.1b1 (HMMs available upon request). HMMsearch was used with these HMMs against all GRAS proteins in order to determine each of the sub families. To aid this selection, HMM search scores were derived of the basalmost sequences of the phylogenetic clade in question, and of the next closest phylogenetic clade in the tree. Cutoff scores were then derived to lie between these values. Because the resulting list of sequences of RAD1 and RAM1 largely overlapped, these two clades were combined in a single phylogenetic analysis. While all sequences of non-vascular plants were used, seed plants were represented by selected species to cover gymnosperms, basal angiosperms as well as mono- and di-cotyledonous flowering plants. Additionally, a putative *Lunularia cruciata* RAM1 sequence (Delaux et al., 2015) was added, but based on our phylogenetic analyses could not be confirmed as RAM1. Each of the three protein sets was aligned using Mafft L-INS-i (Katoh and Standley, 2013). Alignments were manually curated using Jalview (Waterhouse et al., 2009), removing identical sequences and cropping columns to only represent the GRAS domain. Sequences of non-symbiotic GRAS proteins, namely the *A. thaliana* DELLA proteins GAI and RGA, were added for outgroup rooting (see Supplementary Figure S1 for relationships of the GRAS clades). The best suited amino acid substitution model was determined using Prottest 3 (Darriba et al., 2011) and turned out to be JTT+G+F. Bayesian inference utilizing MrBayes 3.2 (Ronquist et al., 2012) was carried out with two hot and cold chains until the average standard deviation of split frequencies was below 0.01 and no more trend was observable. 150 trees each were discarded as burn-in. Resulting trees were visualized using FigTree 1.4.0^1^. The three alignments that are the basis for the phylogenetic trees are provided as Supplementary Files.

Transcriptome completeness was assessed by determining the percentage of eukaryotic single copy orthologs represented as full length transcripts (Supplementary Table S1), as implemented in BUSCO (Simao et al., 2015). The 1KP transcriptome datasets are based on whole plants.

## Results

Many factors of the CSP have been identified in recent years and it has been shown that plants which do not engage in a mutualistic symbiosis with AMF lost CSP genes (Delaux et al., 2014; Favre et al., 2014; Bravo et al., 2016). Furthermore, it was shown that basic factors of the CSP, such as DMI3/CCaMK and DMI1/POLLUX, were already present in the streptophyte algae, the sister lineage of land plants (Wickett et al., 2014; Delaux et al., 2015). Most probably GRAS proteins and other TFs act in a complex way by forming homo- and hetero-dimers (or multimers) among each other which then regulate the respective target genes (Gobbato et al., 2012, 2013; Hohnjec et al., 2015; Xue et al., 2015). Since mycorrhiza establishment requires fundamental changes to cell structure and physiology, the transcriptional program needs to be tightly regulated and factors involved are key regulators of this alteration. This regulatory network was so far predominantly studied in spermatophytes (especially in the legumes lotus and medicago) leading to a somewhat biased knowledge about presence and absence of these factors. Therefore, we screened for homologs of GRAS TFs (NSP1, NSP2, RAM1, RAD1) previously described in the prime model organisms for mycorrhiza research, *Medicago truncatula* and/or *Lotus japonicus*, and further analyzed them by phylogenic inference with special emphasis on bryophytes. Through this we were able to elucidate probable presence/absence patterns for factors of the GRAS transcriptional network in bryophyte clades.

### Presence and Absence of Factors of the GRAS Regulatory Network in Bryophyte Lineages

**FIGURE 2:**
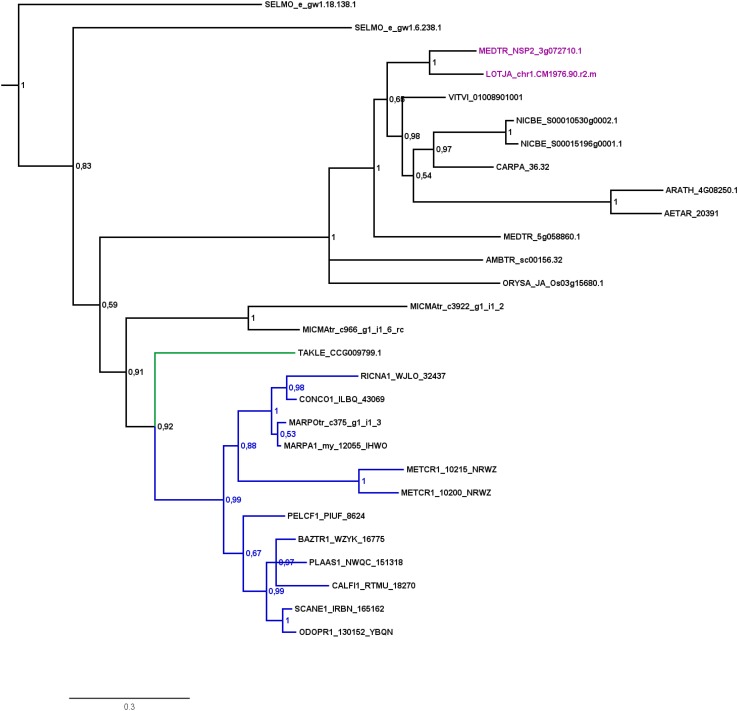
Phylogenetic tree for NSP2. Phylogenetic reconstruction of NSP2 using Bayesian inference. NSP2 was found in the basal lineage represented by the moss *Takakia* (green branches) and in liverworts (blue branches), but not in hornworts. Sequences of *L. japonicum* and *M. truncatula* are highlighted in purple. Five letter codes of the form *MARchantia POlymorpha* (MARPO) are used to abbreviate species names (Supplementary Table S1). Five letter codes followed by “1” depict 1KP sequences, those followed by “tr” represent sequences from non-1KP transcriptomes. The *Marchantia paleacea* transcriptome generated from mycorrhized tissue is marked by “my,” the one generated from non-mycorrhized tissue by “nm.” Note that an NSP2 transcript was found in the mycorrhized (my) 1KP library for *Marchantia paleacea*, as well as in the *Marchantia polymorpha* transcriptome. Posterior probabilities are shown at the nodes, the tree was outgroup-rooted by *A. thaliana* GAI and RGA (not shown).

**FIGURE 3:**
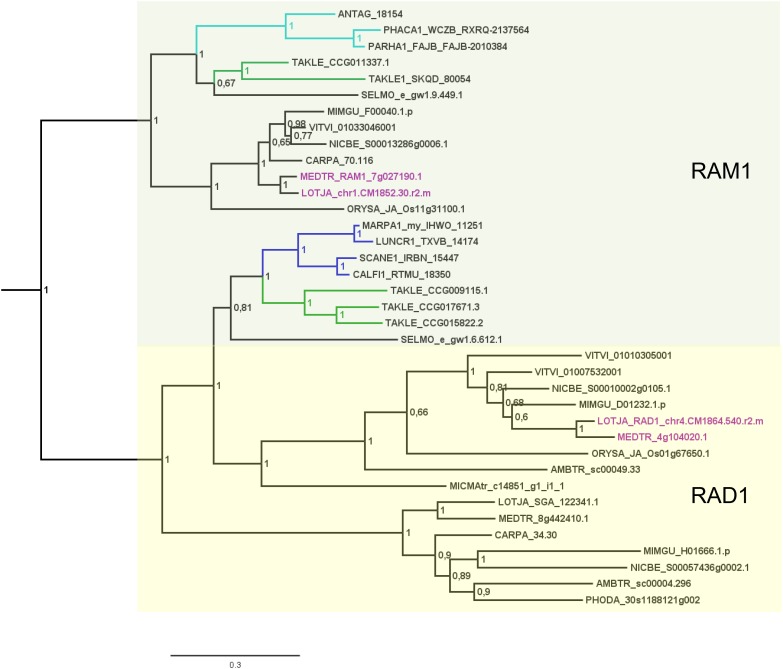
Phylogenetic tree for RAD1 and RAM1. Phylogenetic reconstruction of RAD1 and RAM1 (clades marked using boxes) using Bayesian inference. RAD1 was found in the basal moss lineage represented by *Takakia* (green branches) and in liverworts (blue branches), but not in hornworts. In contrast, RAM1 was found only in *Takakia* and hornworts (cyan branches). Sequences of *L. japonicum* and *M. truncatula* are highlighted in purple. Note that a RAD1 transcript was only found in the mycorrhized (my) 1KP library for *Marchantia paleacea*, and that no RAD1 gene was found in the *Marchantia polymorpha* genome or transcriptome. Posterior probabilities are shown at the nodes, the tree was outgroup-rooted by *A. thaliana* GAI and RGA (not shown); see legend to Figure 2 for explanation of naming.

**FIGURE 4:**
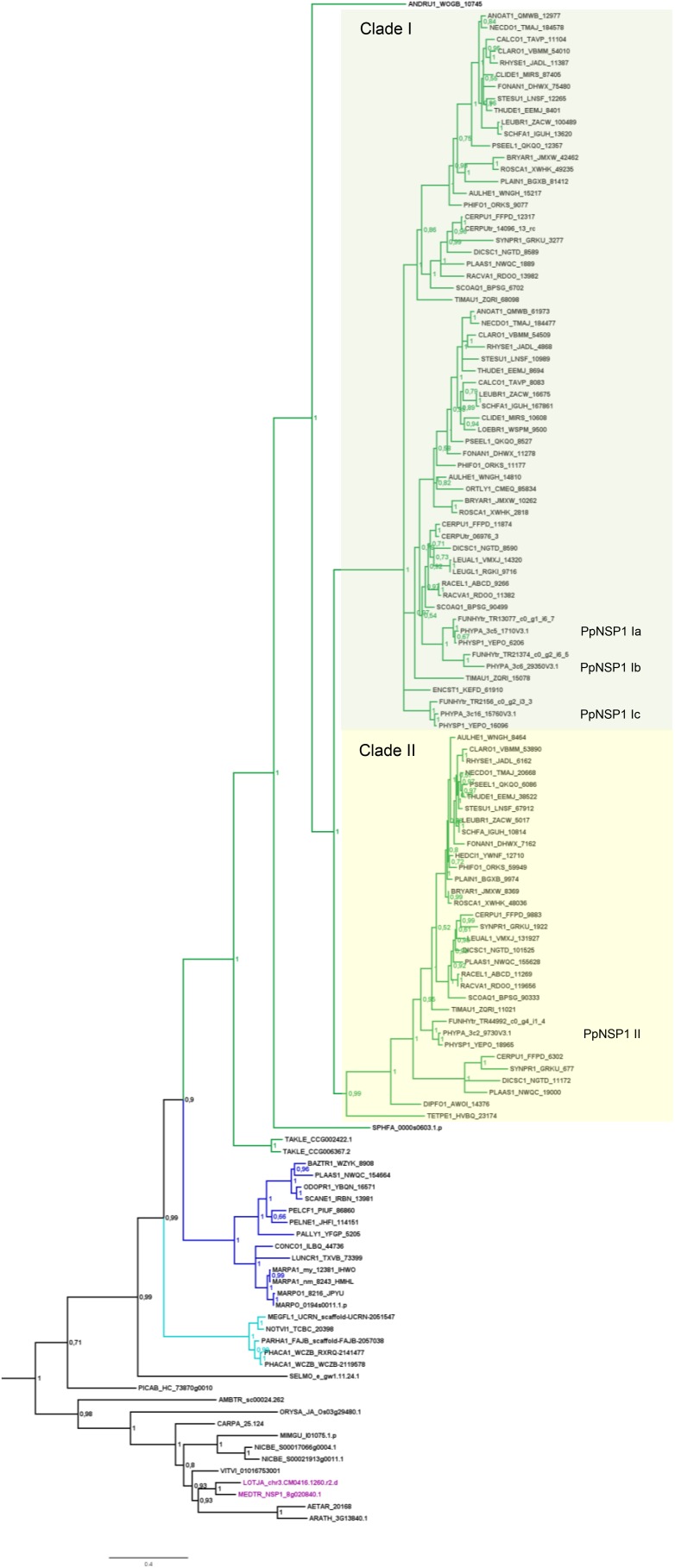
Phylogenetic tree for NSP1. Phylogenetic reconstruction of NSP1 using Bayesian inference. NSP1 was found in mosses (green branches), liverworts (blue branches), and hornworts (cyan branches). In the case of crown group mosses (Bryophytina) an expansion of NSP1 is evident due to the presence of several paralogs per species. Two main clades (marked by boxes, clades I and II) can be observed for moss NSP1. Mosses typically encode four NSP1 copies; naming of individual NSP1 genes is provided for *P. patens* as an example. Note that the basal lineages represented by *Takakia, Sphagnum*, and *Andreaea* do not share the diversified NSP1 clades of the other mosses. Sequences of *L. japonicum* and *M. truncatula* are shown in purple. Note that NSP1 transcripts were found in all transcriptomes of *M. paleacea* and *M. polymorpha*. Posterior probabilities are shown at the nodes, the tree was outgroup-rooted by *A. thaliana* GAI and RGA (not shown); see legend to Figure 2 for explanation of naming.

Analysis of factors downstream of the signaling module and Ca^2+^ spiking (CCaMK) in non-seed plants and algae has in part already been undertaken recently (Delaux et al., 2015). Analyzing specifically the GRAS TFs and creating GRAS TF trees in more depth (Figures 2–4 and Supplementary Figure S1), we found no orthologs of NSP2 (Figure 2), RAD1 and RAM1 (Figure 3) in *P. patens* or other crown group mosses (Bryophytina) based on fully sequenced genomes, transcriptome data (Szovenyi et al., 2010, 2014) and data from the 1,000 plant transcriptomes project 1KP (Matasci et al., 2014). We also included genomic data for the more basal lineages of *Sphagnum* and *Takakia*. In case of *Sphagnum* we were able to detect only NSP1 (Figures 1B, 4). In contrast to that we found all factors to be present in *Takakia*.

Our GRAS phylogenetic analyzes did not detect liverwort orthologs of RAM1, although 1KP data (Matasci et al., 2014) were included (Figure 3). Apart from that we found NSP1, NSP2, and RAD1 in the liverwort lineage. Interestingly, while NSP1 and NSP2 are detected in mycorrhizal as well as non-mycorrhizal tissue of *Marchantia paleacea* and *Marchantia polymorpha*, RAD1 is only detected in mycorrhizal *M. paleacea* (Figure 3). For hornworts we had access to 1KP data and preliminary sequence data for *Anthoceros agrestis* (kindly provided by Peter Szovenyi). We could identify NSP1 and RAM1, but not NSP2 or RAD1 in hornworts (Figures 1–4). In summary, the only full set of symbiotic GRAS TFs in bryophytes was detected in genomic data of the basal moss lineage represented by *Takakia lepidozioides* (kindly provided by Yikun He).

### Lineage-Specific Expansion of GRAS TFs

Besides the mentioned lineage-specific absence of genes, our analyzes show expansions of some GRAS sub families. Although no orthologs for NSP2, RAM1, and RAD1 were found in *P. patens*, we found four paralogs for NSP1 and a general expansion of this GRAS TF in crown group mosses (Bryophytina). Two main clades of NSP1s in mosses are obvious and most mosses seem to possess four NSP1 paralogs divided into the two clades (Figure 4). In case of *Sphagnum* and *Andreaea* we detected only one copy of NSP1 each, and two copies in case of *Takakia*. These three species represent the sister lineages to the Bryophytina and obviously did not share the later evolutionary diversification of NSP1. Interestingly, expansions of the other three GRAS sub families were only observable for *Takakia*, for which we identified one NSP2 but two paralogs of RAM1 and three paralogs of RAD1 (Figure 3).

Taken together, we identified a specific absence/presence pattern for the analyzed GRAS TFs involved in mycorrhiza signaling. Losses of parts of the symbiotic GRAS genes were found in all examined bryophyte lineages except for *Takakia*. *P. patens* as example for the mosses shows most losses, having lost NSP2, RAM1 and RAD1. Expansions of factors were detected in *Takakia* (NSP1, RAM1, RAD1) and Bryophytina (NSP1) (Figure 1B). Although data of algae were included in the database mined, and GRAS TFs are present in streptophyte algae (Bowman et al., 2017; Wilhelmsson et al., 2017), orthologs of the symbiotic GRAS TFs could not be identified in algae.

## Discussion

From the beginning of their conquest of land, around 500 Ma ago (Lang et al., 2010; Morris et al., 2018), land plants most probably have been in contact and/or symbiosis with fungal partners and nowadays over 80% of all extant land plants continue this mutualistic relationship (Fitter, 2005). Plants make use of a signaling module for recognition and establishment of the symbiosis, the main factors of which have already been present before the water-to-land transition of plants (Delaux et al., 2015). This might indicate that these factors are also important for microbial interactions in an aquatic environment, which are also common although so far less studied (Hempel et al., 2008; Rodriguez et al., 2009; Wurzbacher et al., 2010; Kataržytė et al., 2017). This view is supported by the fact that root nodule symbioses also make use of the CSP to establish plant–bacterial symbiosis (Oldroyd, 2013). Additionally, some microbes adopt this pathway to have parasitic access to plants; most probably parasitism is as ancient as symbiosis or predated and led to it (Corradi and Bonfante, 2012; Wang et al., 2012; Gobbato et al., 2013; Rey et al., 2015, 2017). As outlined above, components of the signaling module but not symbiosis related GRAS TFs were already present in the most recent common ancestor of land plants and charophyte algae. Indeed, we were also not able to identify sequences of charophytes orthologous to ‘symbiotic’ GRAS TFs. As proposed before (Delaux et al., 2015) the symbiotic GRAS signaling most probably evolved by duplication events from GRAS TFs already present in streptophyte algae (Bowman et al., 2017; Wilhelmsson et al., 2017).

### Symbiotic GRAS TFs in Bryophytes – Duplications and Losses

Delaux et al. (2015) detected orthologs for symbiotic GRAS TFs in bryophytes. The full complement was detected for liverworts only (especially *Lunularia cruciata*, for which the transcriptome was sequenced). Furthermore, they identified two factors (RAM1 and RAD1) for *Takakia*, and NSP1 in hornworts and mosses. Our GRAS phylogenetic trees (Figures 2–4) expand this view in so far that we found all four factors in *Takakia*, added the basal moss lineage represented by *Sphagnum* (having NSP1 only), and found NSP2 and RAM1 in hornworts. We were not able to identify RAM1 in liverworts. The previously reported putative *Lunularia cruciata* transcriptomic RAM1 sequence (Delaux et al., 2015) grouped outside the RAM1/RAD1 clade in our analysis, maybe due to its fragmentary sequence. The detection of these GRAS TFs in transcriptomic data is potentially flawed because some of them are only expressed upon detection of or colonization by AM fungi, and thereby activation of the CSP (Xue et al., 2015; Pimprikar et al., 2016; Rich et al., 2017). Hence, they might not be expressed under the conditions from which the respective transcriptome was sequenced. Additionally, hornworts are unfortunately underrepresented in the 1KP data. Such problems do not apply if full genomes (with a certain quality) are available (e.g., *P. patens* or the liverwort *M. polymorpha*). The strength of our study is that we use for the first time genomic data for each of the bryophyte lineages, thus at least partially overcoming the limitations of transcriptomic data. However, it also clearly demonstrates that we need more genomic data for bryophytes and other non-seed plants (Rensing, 2017).

In the case of mosses, only NSP1 is present and clearly expanded, exemplified by, e.g., *P. patens* encoding four NSP1 genes. Using the *M. truncatula* sequences encoding NSP2, RAD1 and RAM1, no hits can be recovered in the *P. patens* genome assembly. Also, the best BLASTP hits of the *M. truncatula* genes flanking the three GRAS loci are not part of syntenic regions detected between *P. patens* and other plant genomes (Lang et al., 2018). Neither is any of the four NSP1 paralogs part of such a syntenic region. Hence, the regions encoding three of the four genes seem to have been lost from the genome. As outlined above, there are two moss NSP1 subclades (Figure 4, clades I and II). Most of the mosses have at least one sequence in each of these clades, and typically encode four paralogs. The topology and distribution pattern of NSP1 genes indicate a common duplication event giving rise to the two clades, observed in the crown group mosses (Bryophytina) but not shared by the sister lineages represented by *Takakia, Sphagnum*, and *Andreaea*. These duplications might be related to whole genome duplication (WGD) events observable in mosses (Lang et al., 2018), leading to subsequent neo- and/or sub-functionalization of duplicated genes (Rensing, 2014). Published expression for *P. patens* (Perroud et al., 2018) shows that the four paralogs show different expression levels (Nsp1 Ib lowest, Nsp1 Ia highest), but a qualitatively similar expression profile across the available developmental stages (Supplementary Table S1). NSP1 was identified as being important in root nodule symbiosis (RNS) (Catoira et al., 2000; Smit et al., 2005) and later on it was shown to also influence AM, since this TF is an important factor of the strigolactone (SL) biosynthesis pathway (Liu et al., 2011; Delaux et al., 2013b; Takeda et al., 2013; Hohnjec et al., 2015). SL biosynthesis is important for the establishment of the AM symbiosis because the fungus senses SL and the hyphal branching increases upon this stimulus (Akiyama et al., 2010). Interestingly, although *P. patens* does not encode an NSP2 gene, which is also necessary for SL biosynthesis in seed plants (Liu et al., 2011), it releases SLs (Proust et al., 2011). Biosynthesis of SL in *P. patens* is induced by phosphate starvation (a condition under which AMF association typically occurs), and leads to resistance to pathogenic fungi (Decker et al., 2017). Potentially, duplicated moss NSP1 genes sub-/neo-functionalized and compensate for the loss of NSP2. As GRAS proteins can act as hetero- and/or homodimers (Hirsch et al., 2009; Li et al., 2016) it is a feasible scenario that the four NSP1 paralogs might take over functions typically carried out by other GRAS proteins in other plants. Intriguingly, only NSP1 genes of clade I (Figure 4) seem to have been duplicated in mosses (the naming of individual NSP1 genes in the overview in Figure 1B is according to this division). This might indicate that a first sub- or neo-functionalization of NSP1 genes already occurred after the first duplication and in the second duplication event duplicated genes of group II were selected against, maybe because of unfavorable consequences due to stoichiometry of the dimer partners. However, so far, we are not able to assign certain functions to the individual NSP1 genes in *P. patens*.

### Presence/Absence of Factors Coinciding With the Host/Non-host Status

Looking at the potential overall evolution of the symbiosis GRAS signaling genes we found support for the view of Delaux et al. (2015). Given the distribution of GRAS factors we hypothesize that the land plant ancestor encoded the full set of GRAS TFs (Figure 1). In bryophyte clades we can observe several losses (especially in mosses) and expansions (also mainly in mosses) of these GRAS TFs. Mosses have lost most factors of these GRAS genes (NSP2, RAM1, RAD1) and according to that they, including *Sphagnum*, are considered non-host plants (Read et al., 2000; Wang and Qiu, 2006). Although the signaling module seems to be intact, they also lost some genes which are, e.g., important for the periarbuscular membrane (Wang et al., 2010; Delaux et al., 2014, 2015). The symbiotic GRAS sub family losses might explain why a tight association or even symbiosis cannot be established in mosses. An exception at the basis of the mosses is *Takakia*, which encodes all four GRAS factors (plus expansions for NSP1, RAM1, and RAD1), and indeed was reported to engage in AM (Boullard, 1988). Given that *Takakia* represents one of the sister lineages of the Bryophytina, an evolutionary loss of NSP2, RAD1, and RAM1 during moss evolution appears the most probable scenario.

Most liver- and hornworts are considered host plants (Field and Pressel, 2018). As mentioned, we were not able to identify RAM1 in liverworts, or NSP2 and RAD1 in hornworts. This might be due to the mentioned problem with transcriptome coverage, but is also explainable by species-specific losses of AM capability exemplified by *M. polymorpha*, which does not show AM and lacks RAD1 (Figure 3) in contrast to its close relative *M. paleacea* (Humphreys et al., 2010; Bowman et al., 2017). The lack of *M. polymorpha* mycorrhizal association is in line with the absence of GAs and might be featured by nutrient rich habitats (Ligrone et al., 2007). While genes needed for successful mycorrhization are absent in non-host *Marchantia* species, other gene families are over-represented in *M. polymorpha*, e.g., transporters for phosphate and ammonium. These genomic adaptations might reflect the shift from mycorrhizal to non-mycorrhizal status by improving the transport capacity instead of being dependent on symbiotic organisms (Bowman et al., 2017). Nevertheless, according to our analysis both lack RAM1, which is not in line with the species’ host/non-host status, since *M. paleacea*, as host, should have the complete GRAS gene set. This is most probably due to the mentioned incomplete nature of the transcriptomic data. In case of NSP2 we can identify a potential coding region in the *M. polymorpha* genome (encoding the same protein detected in the transcriptome, Figure 2) that does not have a gene model assigned to it; updated genome versions might solve this issue. Presence of transcripts in transcriptomic data is evidence of presence of the gene, but absence of transcripts must not necessarily reflect absence of the gene (for example, the 1KP transcriptomes contain on average 84% of the conserved eukaryotic single copy gene set, Supplementary Table S1). For our overview (Figure 1B) we are thus relying mainly on genomic data in order not to represent conclusions based on transcriptomic absence of genes.

### Functions, Additional Factors and Evolution of the Symbiotic Pathway

With rising morphological complexity more complex regulation and cellular reorganization is needed. Most probably we do not yet know all TFs involved in the regulation of this symbiosis, which involves tight regulation and massive cellular reorganization. Recent publications indicate that even more factors are involved, at least in seed plants (Xue et al., 2015; Heck et al., 2016). This indicates that we are only at the beginning of understanding this complex pathway and its transcriptional network (Genre and Russo, 2016; Pimprikar and Gutjahr, 2018). However, a quick survey showed that for example the GRAS TF MIG1 seems not to be present in bryophytes (data not shown). Most probably the transcriptional network to establish mycorrhizal symbiosis comprises more factors and is thereby more complex in vascular plants, due to more cell types and tissue layers. It is important to note that our current knowledge of the CSP is predominantly based on studies in spermatophytes (and here again predominantly analyzed of lotus and medicago). Maybe some GRAS genes are less important in bryophytes as compared to the situation in seed or vascular plants, for example due to their lack of roots.

If we evaluate the evolution of the CSP and its downstream components we should also be considering other plant–microbe associations and symbioses. Recently, the view was broadened since fungi belonging to the Mucoromycotina and also Ascomycotina were shown to interact in particular with liverworts and maybe hornworts (Field et al., 2014; Kowal et al., 2018), and the plant–Mucoromycotina interaction was proposed to potentially represent the ancestral state of plant–fungus interaction (Field et al., 2015). It is also known that plants interact with bacteria or even with both, fungi and bacteria (Bonfante and Anca, 2009). Foremost known is the RNS, which makes use of many factors of the CSP (Oldroyd, 2013; Genre and Russo, 2016). Interaction with cyanobacteria is also known, in particular for hornworts but also in some liverworts, mosses, ferns, and seed plants. There are more microbial/plant associations and symbioses known (e.g., *Frankia*, etc.) (Santi et al., 2013; Martin et al., 2017), and most probably many more we do not know yet. These interactions are important and widespread, and probably evolved already in the aquatic environment (Croft et al., 2005; Hom and Murray, 2014). How are these associations and symbioses regulated and how do the symbiotic partners identify each other? Most likely key components of the CSP (signaling module) are also involved in symbioses other than the ones they have so far been implicated in (mycorrhizal, rhizobial, and actinorhizal) (Martin et al., 2017). The signaling module apparently evolved in streptophyte algae (Delaux et al., 2015), suggesting that it may be functional in additional associations and symbioses, e.g., with cyanobacteria. The factors that process the symbiotic calcium spiking and induce a specific transcriptional program might be specific for each kind of symbiosis, leading to an association/symbiosis-specific diversification of the CSP downstream of the signaling module. More molecular studies in additional symbioses, especially in aquatic environments, are needed to unravel additional symbiosis-specific factors.

## Conclusion

The key pathway to regulate beneficial interactions in plants seems to be the CSP (Martin et al., 2017). Furthermore, it is believed that microbial interactions enabled plants to conquer the land (Pirozynski and Malloch, 1975), highlighting the importance of this signaling pathway. Here we argue that the symbiosis related GRAS signaling genes, known to be important in regulation of AM symbiosis, were already present in the most recent common ancestor of all land plants. These genes display lineage specific losses and expansions in bryophytes, in particular in mosses. Such losses seem to reflect the non-host status. Nevertheless, the upstream CSP signaling module for symbiosis establishment seems to be intact (Wang et al., 2010; Delaux et al., 2015) and may serve in additional symbioses with a different or an extended subset of factors in the transcriptional network module. Additional studies are needed to elucidate the symbiosis-specific interplay of TFs and the functions of, e.g., the duplicated NSP1 genes in mosses.

## Author Contributions

SAR and CG conceived of the study. SAR carried out the phylogenetic analyses. CG, ACG, and SAR analyzed the data. CG and SAR wrote the paper, with contributions by ACG.

## Conflict of Interest Statement

The authors declare that the research was conducted in the absence of any commercial or financial relationships that could be construed as a potential conflict of interest.

## Footnotes

1 http://tree.bio.ed.ac.uk/software/figtree

## Supplementary Material

FIGURE S1 Overview phylogeny of the GRAS family. Midpoint rooted Bayesian inference tree of selected species, including *Lotus japonicus, Medicago truncatula, Arabidopsis thaliana, Physcomitrella patens*, and *Marchantia polymorpha*. Line thickness corresponds to posterior probabilities. Colored clades depict RAD1 (red), RAM1 (purple), NSP1 (blue), NSP2 (green), and DELLA (cyan). Note that DELLA proteins were used as outgroup to root each of the trees shown in Figures 2–4.

TABLE S1 Lists the sources of genomic and transcriptomic datasets used, five letter species abbreviations used in the Figures, BUSCO completeness percentages and expression data for the *P. patens* NSP1 paralogs. Alignments of NSP1, NSP2, and RAD1/RAM1 used for the phylogenetic analyses shown in Figures 2–4.
