# Supplementary material for: Evolution of the Symbiosis-Specific GRAS Regulatory Network in Bryophytes
Source: Front Plant Sci. 2018 Nov 6;9:1621. doi: 10.3389/fpls.2018.01621 (PMC6232258; doi:10.3389/fpls.2018.01621)
Supplement: FIGURE S1 — Overview phylogeny of the GRAS family. Midpoint rooted Bayesian inference tree of selected species, including Lotus japonicus, Medicago truncatula, Arabidopsis thaliana, Physcomitrella patens, and Marchantia polymorpha. Line thickness corresponds to posterior probabilities. Colored clades depict RAD1 (red), RAM1 (purple), NSP1 (blue), NSP2 (green), and DELLA (cyan). Note that DELLA proteins were used as outgroup to root each of the trees shown in Figures 2–4. [file Image_1.pdf]

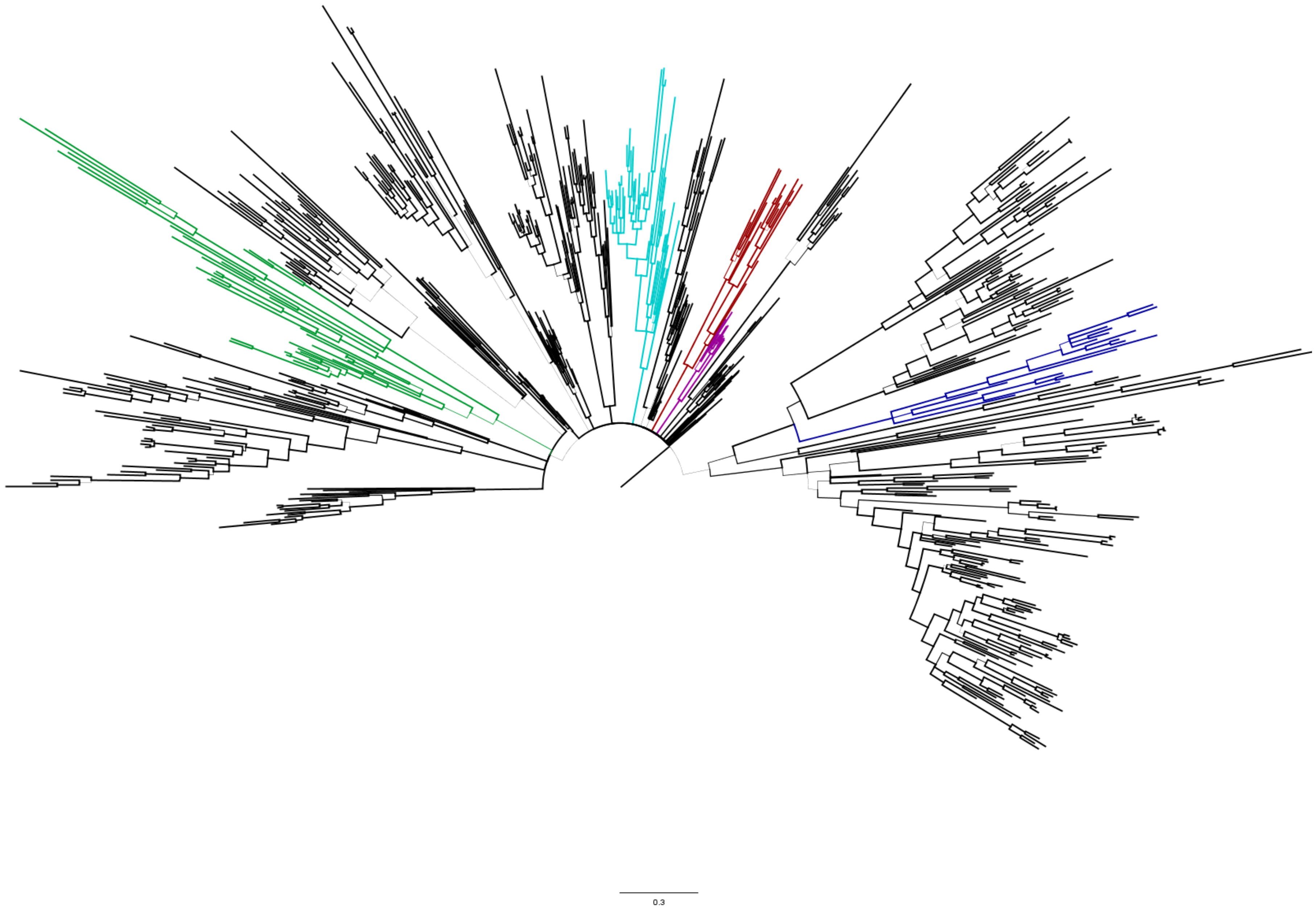
0.3

Fig. S1: Overview phylogeny of the GRAS family.
Midpoint rooted Bayesian inference tree of selected species, including *Lotus japonicus*, *Medicago truncatula*, *Arabidopsis thaliana*, *Physcomitrella patens* and *Marchantia polymorpha*. Line thickness corresponds to posterior probabilities. Colored clades depict RAD1 (red), RAM1 (purple), NSP1 (blue), NSP2 (green) and DELLA (cyan). Note that DELLA proteins were used as outgroup to root each of the trees shown in Fig. 2-4.
